# Shear stress attenuates apoptosis due to TNFα, oxidative stress, and serum depletion via death-associated protein kinase (DAPK) expression

**DOI:** 10.1186/s13104-015-1037-8

**Published:** 2015-03-18

**Authors:** Keith Rennier, Julie Y Ji

**Affiliations:** Department of Biomedical Engineering, Indiana University Purdue University Indianapolis, 723 West Michigan Street, SL-220 J, Indianapolis, IN 46202 USA

**Keywords:** Shear stress, DAPK, Apoptosis, TNFα

## Abstract

**Background:**

Misdirected apoptosis in endothelial cells participates in the development of pathological conditions such as atherosclerosis. Tight regulation of apoptosis is necessary to ensure normal cell function. The rate of cell turnover is increased at sites prone to lesion development. Laminar shear stress is protective against atherosclerosis, and helps suppress apoptosis induced by cytokines, oxidative stress, and serum depletion. Current Studies have shown that the pro-apoptotic DAPK expression and function to be regulated in part by shear stress, and that shearing cells already treated with cytokine tumor necrosis factor (TNF) α significantly reduced apoptosis. We investigate further the suppression of endothelial apoptosis by shear stress with other apoptotic triggers, and the involvement of DAPK and caspase 3/7.

**Results:**

We have shown that exposure to shear stress (12 dynes/cm^2^ for 6 hrs) suppressed endothelial apoptosis triggered by cytokine (TNFα), oxidative stress (H_2_O_2_), and serum depletion, either before or after a long term (18 hr) induction. This is correlated with a parallel decrease of DAPK expression and caspase activity compared to non-sheared cells. We found similar modulation of DAPK and apoptosis by shear stress with other pro-apoptotic signals. Changes in DAPK and caspase 3/7 are directly correlated to changes in apoptosis. Interestingly, shear stress applied to cells prior to induction with apoptosis agents resulted in a higher suppression of apoptosis and DAPK and caspase activity, compared to applying shear stress post induction. This is correlated with a higher expression and activation of DAPK in cells sheared at the end of 24-hr experiment. Also, shear stress alone also induced higher apoptosis and DAPK expression, and the effect is sustained even after 18 hrs incubation in static condition, compared to non-sheared cells.

**Conclusions:**

Overall, we show that laminar shear stress inhibits various apoptosis pathways by modulating DAPK activity, as well as caspase activation, in a time-dependent manner. Shear stress could target DAPK as a converging point to exert its effects of suppressing endothelial apoptosis. The temporal shear stress stimulation of DAPK and its role in different apoptosis pathways may help identify key mechanisms of the endothelial mechanotransduction pathway.

## Background

Endothelial cells that line the lumen of blood vessels are continuously exposed to hemodynamic forces such as shear stress on the apical side and circumferential stretch on the basal side due to blood flow. Shear stress, the result of velocity gradient and blood viscosity, acts parallel to endothelial surface, and disturbances in the vasculature, such as bending and bifurcation, give rise to different localized magnitude and direction of shear stress. Shear stress is shown to be an important regulator of endothelial functions [1]. Regions of disturbed flow with reversing or vortexing flow are prone to developing atherosclerosis while sections of uniform laminar flow tend to protect against atherosclerosis. While apoptosis or programmed cell death is important for maintaining homeostasis, increased cell turnover exists at sites of low shear [2,3], and uniform shear stress acts as a potent inhibitor of apoptosis induced by serum depletion, oxidative stress, and cytokine (TNF) α [4-6]. Understanding apoptosis activation in response to fluid flow effects may further elucidate shear stress activated cellular mechanisms in both physiological and pathological conditions.

Endothelial apoptosis is influenced by a wide variety of signal transduction pathways. Shear stress due to fluid flow can activate or inhibit endothelial apoptosis depending on the environmental factors and local tissue-fluid flow interaction [7]. While some apoptosis signaling cascades have been investigated, signal transduction pathways in endothelial apoptosis under fluid shear stress are not fully understood. Most *in vitro* studies on identifying key molecules of apoptosis signaling were not done in the presence of shear stress [8-12]. On the other hand, several shear stress studies that examined inflammatory protein expression did not quantify the subsequent endothelial apoptosis [13-17].

Recent research has shown that death-associated protein kinase (DAPK) is a positive mediator for apoptosis [18]. DAPK is a 160 kDa Ca^2+^ calmodulin (CaM)-dependent serine/threonine protein kinase that is triggered due to various stimuli including TNFα, interferon (IFN-γ), ceramide and oncogenes such as p53 [19-23]. DAPK contains a CaM binding domain, a cytoskeleton binding domain, eight ankyrin repeats, two P-loops which is a putative nuclear binding domain, plus an independent death domain necessary for apoptosis initiation [24]. Auto-phosphorylation of DAPK at serine 308 in the Ca^2+^/CaM binding domain, in normal cells, is an important inhibitory regulatory checkpoint [25]. Dephosphorylation of serine 308 occurs following apoptotic signals trigger, which along with Calmodulin binding are required for complete activation of DAPK and its catalytic activities.

Besides the key role in apoptosis, DAPK also contributes to cytoplasmic changes linked to apoptosis, such as stress fiber development and membrane blebbing. DAPK is localized to the actin extracellular network where it regulates actin and cytoplasm changes associated with programmed cell death [19,24,26]. Under fluid shear, endothelial cells introduce stress fiber formation and focal adhesion re-alignment. As a result, the morphological changes align the cell cytoskeleton in the direction of fluid shear [27,28]. DAPK in the actin cytoskeletal network could potentially play a role in re-organization of the cytoskeleton.

Current research has mainly focused on DAPK function in select types of cancer [29], but its endothelial function is still being defined. Under hemodynamic shear stress, mechanisms of DAPK regulation in apoptosis and its functions in endothelial cells are largely unknown. Galbraith *et al.* showed that sheared endothelial cells undergo various structural changes. Endothelial cells after long-term shearing facilitate cytoskeletal remodeling, stress fiber formation, increased focal adhesion activity, and eventually realignment with the flow field direction [28,30]. Endothelial cells respond to fluid shear stress by initiating various signal transduction pathways. The DAPK regulatory role in programmed cell death and its interaction with cytoskeletal changes suggest a potential role in endothelial mechanotransduction.

Our recent *in vitro* study explored the effects of fluid shear stress on endothelial DAPK expression [31]. Shear stress alone transiently up-regulated DAPK gene transcription and protein activity, leading to increased apoptosis. However, we identified that shear stress exposure to cells already activated by TNFα substantially decreased the populations of apoptotic cells. We found excellent correlation between DAPK expression and activity as well as in our apoptosis results. On the other hand, DAPK expression knocked-down through siRNA transfection caused increased TNFα-induced apoptosis as compared to the control siRNA and non-transfected endothelial cells. These data suggest a functional role for DAPK due to shear involved in attenuating cytokine (TNFα) triggered apoptosis [31]. Shear stress was shown to modulate DAPK activity while diminishing TNFα induced apoptosis. This study suggests that shear stress can not only generate pro-apoptotic signaling through DAPK activation, but also modulates DAPK expression to protect against endothelial apoptosis in the presence of TNFα [31].

Based on our data that cytokine and shear stress regulated DAPK expression, we further examine subsequent time-dependent effects of shear stress on cytokine-induced apoptosis, as well as other environmental stimulants. Here we explore endothelial apoptosis due to biochemical activators of apoptosis, such as oxidative stress or serum deprivation, and the mechano-sensitive role of DAPK via fluid shear stress. We also examine the competing pathways of shear and apoptosis activation in a time-dependent study. The temporal activation of DAPK and its connected pathway could play a vital role in endothelial response to shear. We continue to clarify the effect of shear stress on DAPK activation in the presence of other apoptotic inducers, through a pre- or post-shear specific study. These studies further explore the impact of DAPK in the endothelial mechanotransduction pathway.

## Results

### Time dependent study on apoptosis and DAPK under shear stress and biochemical stimuli

**Figure 1:**
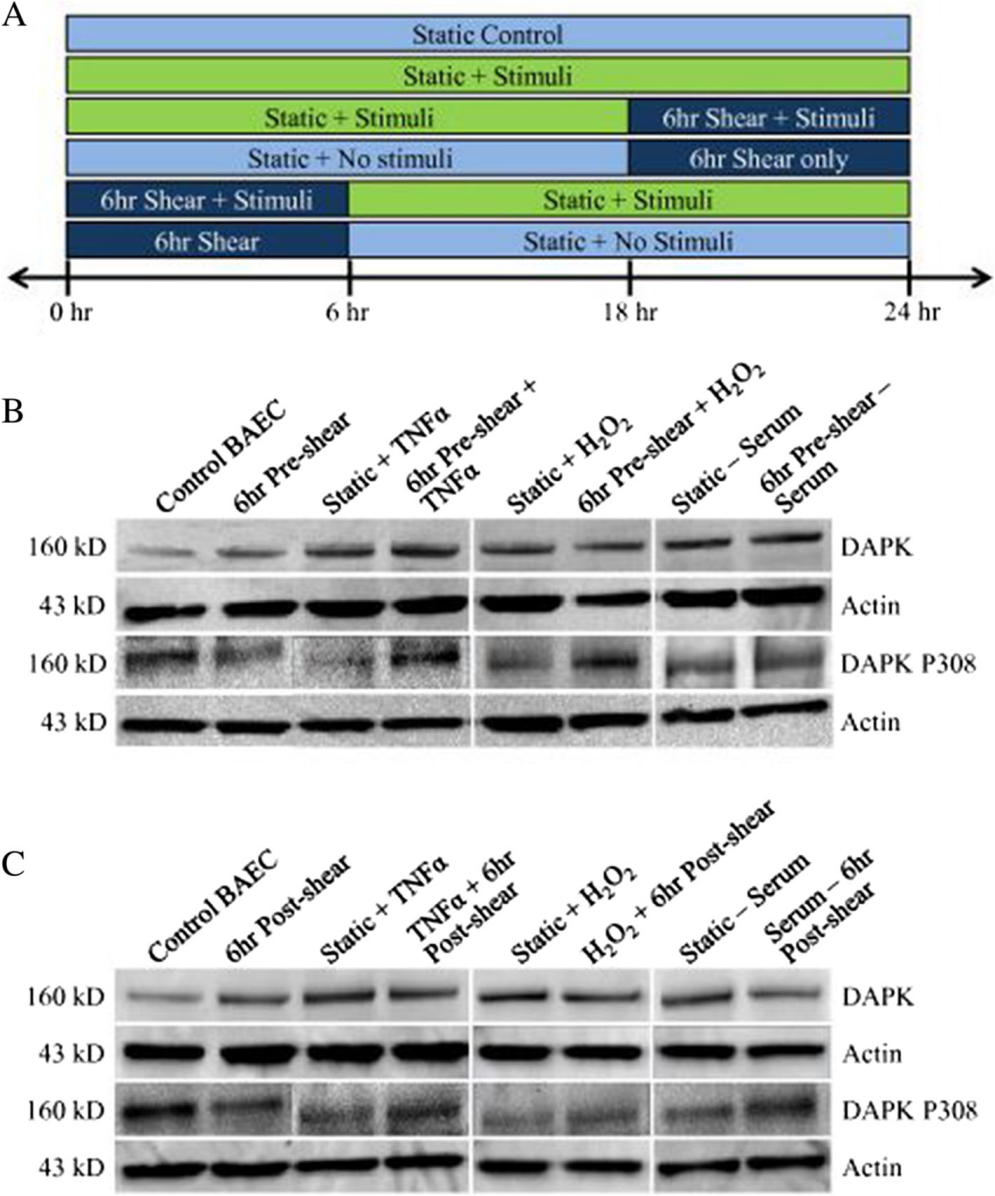
**Experimental design setup and representative DAPK Western blots. A**: Subset design to analyze the effects of simultaneous shear stress and stimuli. Cells are treated to shear stress either before or after treatment with apoptotic stimuli, which includes TNFα, H_2_O_2_, and serum depletion. **B**: Western blot of DAPK in pre-sheared cells versus cells treated with stimulus (TNFα, H_2_O_2_, or serum depletion). **C**: Western blot of DAPK in cells sheared post treatment with stimulus (TNFα, H_2_O_2_, or serum depletion).

We examined the time-dependent effect of shear stress on apoptosis and DAPK expression following stress stimulus. Since our preliminary data showed increased DAPK and apoptosis level after 6 hr shearing alone, we decided to incorporate 6 hr shearing either before or subsequent to apoptotic stimulus, to fully evaluate its effect on DAPK and apoptosis. Cells were categorized as either exposed to shear stress before or after stimulation with an apoptotic trigger. Experiments were carried out based on the following 6 groups: Control BAEC; static incubation with stimulus for 24 hrs (Static + stimulus); 6 hr shear stress then incubation in regular media for 18 hrs (6 hr Pre-shear); 6 hr exposure to shear stress first followed by application of the stimulus for 18 hrs (6 hr Pre-shear + stimulus); incubation with the stimulus for 18 hrs followed by 6 hr shear stress (stimulus + 6 hr Post-shear); and static condition followed by 6 hr post-shear (6 hr Post-shear). They are also represented in the diagram in Figure 1A. For biochemical apoptotic triggers, we chose either cytokine (TNFα treatment at 25 ng/ml), oxidative stress (0.5 mM H_2_O_2_), or serum depletion (low level 0.5% FBS), as the “stimulus” for apoptosis. We will apply the same 24 hr time frame to all groups to capture apoptotic and DAPK results (Figure 1A).

### DAPK expression is decreased in TNFα, H_2_O_2_ treated, and serum depleted cells when exposed to shear stress before or after treatment

**Figure 2:**
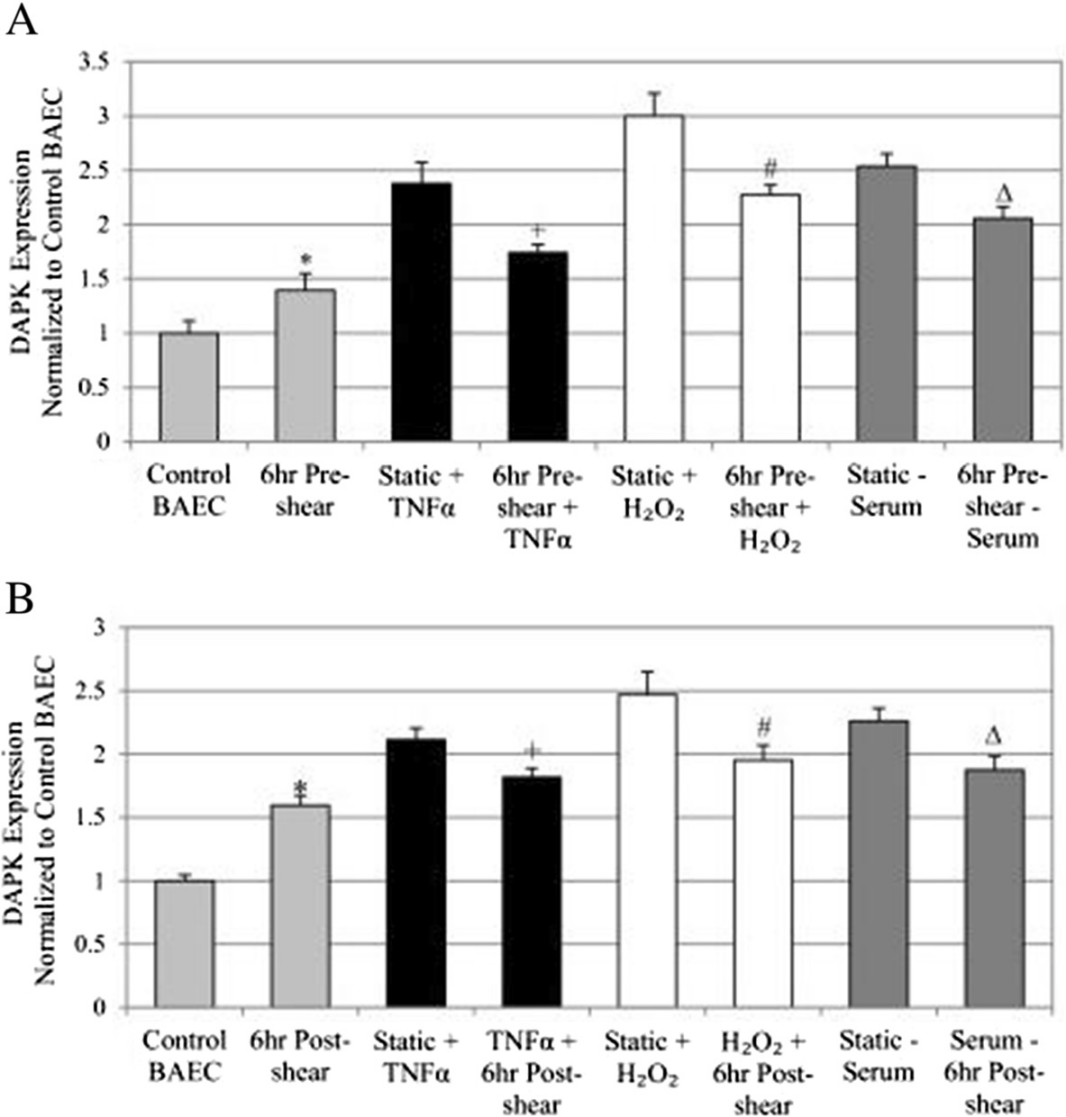
**Overall DAPK expression for pre- and post-sheared experimental groups. A**: DAPK protein expression for the treatment and pre-sheared experimental groups. **B**: DAPK protein expression for the treatment and post-sheared experimental groups. For all figures: * *P* < 0.05 compared to Control BAEC, + *P* < 0.05 compared to Static + TNFα, # *P* < 0.05 compared to Static + H_2_O_2_, ∆ *P* < 0.05 compared to Static – Serum.

First, we explored the effect of pre-conditioning cells with shear stress exposure (12 dynes/cm^2^) in samples with and without each of the stimuli (TNFα, H_2_O_2_, and serum depletion). Cell lysates from each group were collected and analyzed for overall DAPK expression in Western blots (Figure 1B). Samples were duplicated in three independent experiments, and bands were quantified and analyzed for statistical comparison (Figure 2A). Western analysis showed DAPK activity significantly decreased in cells exposed to pre-shearing followed by stimuli incubation compared to stimulants alone (static + stimulus) (Figure 1B). DAPK expression was quantified using the relative intensity of DAPK bands normalized to a loading control, actin (Figure 2A). After quantitative analysis, we consistently observed a statistically significant increase in DAPK expression after exposure to TNFα, H_2_O_2_, or serum depletion, as compared to control BAEC (*P* < 0.05). Also, we saw a significant decrease in DAPK expression in cells pre-sheared prior to stimuli incubation, compared with the static cells exposed to each stimuli (*P* < 0.05). The decrease due to shear stress is observed independent of the apoptosis trigger, which all activated DAPK expression. Based on our quantitative analysis of Western bands, the addition of shear stress before apoptosis trigger decreased DAPK by 27% for TNFα, 24.33% for H_2_O_2_, or 18.58% for serum depletion. Similarly, for the post-sheared experiments, BAEC cells were incubated with each stimulus for 18 hrs prior to 6 hr shear stress. Western blots were carried out to evaluate DAPK expression (Figure 1C), and quantified using the relative band intensity compared to actin (Figure 2B). Again, DAPK expression significantly increased for apoptosis-induced static cells. With the addition of shear stress following apoptosis induction, we observed significant decrease in DAPK expression, compared to cells incubated with stimulants alone (*P* < 0.05). Cells sheared for 6 hrs after exposure to TNFα, H_2_O_2_, or serum depletion still resulted in an increase of total DAPK expression, but less significantly than static cells under the same stimulus conditions (Figure 2B). However, the decrease in DAPK expression by post-induction shear stress was less than that of pre-shearing before apoptotic induction. Based on our quantitative Western analysis, the addition of shear stress after apoptosis trigger decreased DAPK by 14.05% for TNFα, 22% for H_2_O_2_, or 17.22% for serum depletion. Overall, our data suggest that exposure to shear stress mitigates the increase in endothelial DAPK expression brought on by the addition of cytokine TNFα, H_2_O_2_, and serum depletion on compared to the static induction cases.

### Phosphorylated serine 308 DAPK is decreased in TNFα, H_2_O_2_, and serum depleted and shear stress treated endothelial cells

**Figure 3:**
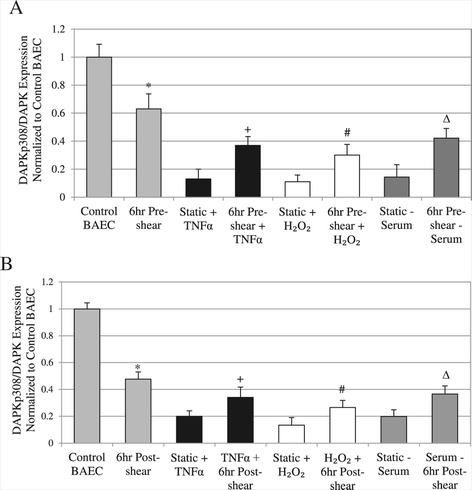
**Phosphorylated DAPK for pre- and post-sheared experimental groups. A**: Ratio of DAPKp308/Total DAPK protein expression for each experimental group, cells treated with TNFα, H_2_O_2_, or serum depletion versus pre-sheared exposure. **B**: Ratio of DAPKp308/Total DAPK protein expression for the treated and post-sheared experimental groups. For all figures: * *P* < 0.05 compared to Control BAEC, + *P* < 0.05 compared to Static + TNFα, # *P* < 0.05 compared to Static + H_2_O_2_, ∆ *P* < 0.05 compared to Static – Serum.

As an inhibitory checkpoint, DAPK is auto-phosphorylated at serine 308 in its inactive state. When DAPK is dephosphorylated at serine 308, in the presence of calmodulin binding, DAPK becomes fully activated for its kinase function. The experimental setup previously described was used to investigate the effect of time-dependent shear stress on phospho-serine 308 DAPK (DAPK P308) in order to assess the level of DAPK activation in addition to expression changes. Western blots are duplicated in independent experiments, and bands were quantified and analyzed for statistical comparison. The values for overall phosphorylated DAPK (DAPK P308) are presented as fractions of the total DAPK within each experimental group (Figure 3). We found that fraction of DAPK P308 decreased after exposure to shear stress, but the decrease was significantly more after treatment with TNFα, H_2_O_2_, or serum depletion in static condition (*P* < 0.01). The decrease in phospho-308 DAPK in cells indicated an increased DAPK activation, and shear stress alone was not as effective as other environmental stimuli in activating DAPK activity. Furthermore, adding apoptotic stimulus after pre-shearing significant increased phospho-308 DAPK in treated cells compared to control with no pre-shearing (*P* < 0.05), indicating a decrease in activated DAPK (Figure 3A). Fraction of phospho- to total DAPK increased by 2.84 fold for TNFα, 2.72 fold for H_2_O_2_, or 2.95 fold for serum depletion.

Similar trends in phospho-308 to total DAPK ratio were observed for cells that were sheared for 6 hrs after 18 hr induction with each stimulus (Figure 3B). Significant increases in phospho-DAPK compared to each apoptotic treatment alone were again observed, suggesting that shear stress also attenuates DAPK dephosphorylation subsequent to and in the presence of long term apoptosis induction (*P* < 0.05). In contrast to the pre-shearing cases, the fractions of phospho- to total DAPK increased to a less extent: 1.79 fold for TNFα, 1.98 fold for H_2_O_2_, or 1.92 fold for serum depletion. Again, these data suggest that adding shear stress reduces the expression and the activation of DAPK in conjunction with apoptotic stimulus such as TNFα, H_2_O_2_, or serum depletion.

### Shear stress significantly reduced apoptosis activated by TNFα, H_2_O_2_, and low serum

Since our data showed that (1) fluid shear stress alone increased DAPK expression and activity, but not to the extent of other apoptotic trigger such as TNFα, H_2_O_2_, or serum depletion; and (2) while each apoptotic trigger activates DAPK expression and activation, both are significantly suppressed by exposure to shear stress either before or after treatment. We further explore the time-dependent effect of shear stress on TNFα, H_2_O_2_, and serum depletion induced endothelial apoptosis.

**Figure 4:**
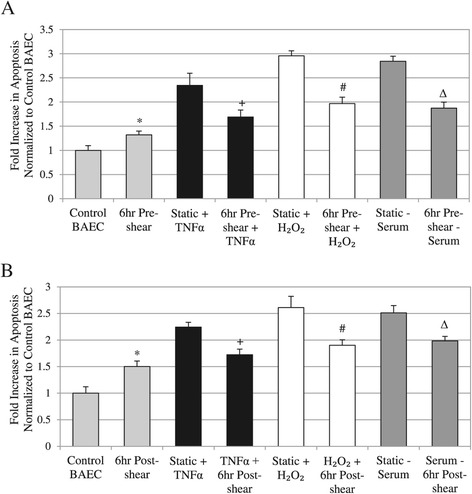
**Quantified cell apoptosis results using TUNEL staining. A**: Analysis of TUNEL in pre-sheared cells versus cells treated with stimulus (TNFα, H_2_O_2_, or serum depletion). **B**: Analysis of TUNEL results in cells sheared post treatment with stimulus (TNFα, H_2_O_2_, or serum depletion). For all apoptosis data: * *P* < 0.05 compared to Control BAEC, + *P* < 0.05 compared to Static + TNFα, # *P* < 0.05 compared to Static + H_2_O_2_, ∆ *P* < 0.05 compared to Static – Serum.

We added TNFα (25 ng/ml), H_2_O_2_ (0.5 mM), and serum depletion (0.5% FBS) to induce apoptosis, in combination with shear stress conditioning (12 dynes/cm^2^). Our apoptosis study combined long term inductions (18 hrs) with either pre-shearing or subsequent post-shearing for 6 hrs, to examine the suppression of apoptosis due to shear stress. Apoptosis was analyzed by TUNEL staining, quantified using flow cytometry, and the fold increase in apoptosis for each sample is calculated (Figure 4). Each apoptotic stimulus induced an approximately two-fold increase in endothelial apoptosis compared to control BAECs under static condition. Fold induction of apoptosis ranged from 2.24 to 2.96-fold increase. Conversely, shear stress exposure either before or after cytokine or biochemical induction significantly decreased overall apoptosis compared to static cells under the same conditions (Figure 4).

Pre-treating cells with shear stress seemed to suppress overall apoptosis induced by TNFα, H_2_O_2_, and serum depletion. Pre-shearing cells in media containing the same apoptotic condition for 6 hrs significantly decreased apoptosis at the end of 24-hr induction period, based on our TUNEL staining with flow cytometry (Figure 4A). There was a similar trend seen when cells were treated to apoptotic trigger for 18 hrs then subsequently sheared for 6 hrs and assessed with TUNEL staining (Figure 4B). Shearing cells after incubation with TNFα, H_2_O_2_, and serum depletion was shown to significantly suppress apoptosis compared to static and treated samples.

Based on our apoptosis analysis, pre-shearing cells before stimulus treatment decreased apoptosis by: 28% for TNFα, 33.51% for H_2_O_2_, or 34.24% for serum depletion compared to static cells (Figure 4A). On the other hand, addition of shear stress after apoptosis stimulus decreased apoptosis by 23.56% for TNFα, 27.18% for H_2_O_2_, or 21% for serum depletion (Figure 4B). Our data showed that adding shear stress post-induction with apoptotic trigger was not as effective in reducing apoptosis as shearing cells prior to adding apoptotic trigger, which seems to suggest a time-dependence in the anti-apoptotic effects of shear stress relative to the length of other biochemical induction of apoptosis.

### Shearing cells before or after apoptosis induction also significantly decreased caspase activity

**Figure 5:**
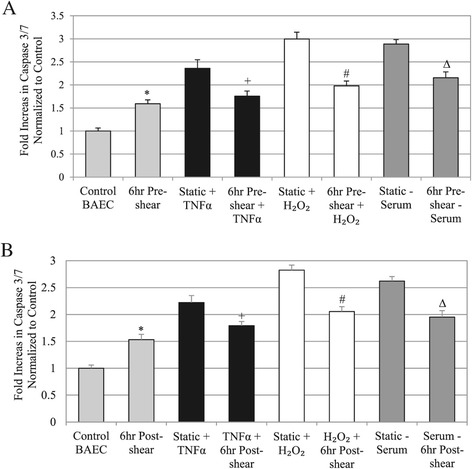
**Quantified cell apoptosis results based on caspase activity. A**: Analysis of Caspase-3 and −7 activity in pre-sheared cells versus cells treated with stimulus alone (TNFα, H_2_O_2_, or serum depletion). **B**: Analysis of Caspase-3 and −7 activity in cells sheared post treatment with stimulus versus cells treated with stimulus alone (TNFα, H_2_O_2_, or serum depletion). For all caspase data: * *P* < 0.01 compared to Control BAEC, + *P* < 0.01 compared to Static + TNFα, # *P* < 0.01 compared to Static + H_2_O_2_, ∆ *P* < 0.01 compared to Static – Serum.

To confirm the TUNEL stain apoptosis results, we also examined caspase activity in each sample (Figure 5). Caspase activity is downstream from DAPK in the apoptosis pathway, and would further link DAPK activity to apoptosis under each treatment. We have shown previously that corresponding caspase 3/7 activity is related to shear regulated DAPK activation [31,32]. Using the same experimental groups, we found that pre-shearing cells similarly decreased caspase activity when compared to each of the stimulant alone treatments (Figure 5A). This data correlate well to our TUNEL results (*P* < 0.01), further confirming activation of the apoptotic pathway. Shearing cells after apoptosis induction also mitigated the overall increase in caspase activity when compared to the static cells exposed to apoptosis inductors (*P* < 0.01) (Figure 5B). Although, shearing cells alone for 6 hrs, without induction with apoptotic trigger, induced a considerable increase in apoptosis and caspase production compared to control cells (no shearing or stimulus) (Figures 4 and 5). These results correlate with our previous discovery that shear stress induced an increase in overall DAPK and decrease of phospho-DAPK, which promotes DAPK activity and subsequent apoptosis.

Pre-shearing cells before stimulus treatment decreased caspase 3/7 activity by: 25.58% for TNFα, 34% for H_2_O_2_, or 25.86% for serum depletion compared to static cells (Figure 5A). On the other hand, addition of shear stress after apoptosis stimulus decreased caspase 3/7 activity by 19.28% for TNFα, 27.05% for H_2_O_2_, or 24.61% for serum depletion (Figure 5B). Again, our data showed that adding shear stress post-induction with apoptotic trigger was not as effective as shearing cells prior to adding apoptotic trigger in reducing caspase 3/7 activity. Thus, we found corresponding caspase 3/7 activity in each experimental group that further support apoptosis results based on TUNEL.

## Discussion

Laminar shear stress suppresses endothelial apoptosis in the presence of various types of inducers such as cytokines (TNFα), ceramide, and other stimulants [4-6]. DAPK’s involvement in endothelial cells has yet to be fully elucidated. Due to key involvement in both apoptosis and cytoskeletal remodeling, and its presence in the actin network, DAPK could play a role in endothelial mechanotransduction regulated by shear stress. Our previous study showed for the first time that shear stress affects DAPK activation, as well as apoptosis following TNFα treatment [31,32], suggests that shear stress uses DAPK as a mechanism of exerting its protective effect against endothelial apoptosis. Since our preliminary data showed increased DAPK and apoptosis level after 6 hr shearing alone, we decided to incorporate 6-hr shearing either before or subsequent to apoptotic stimulus, to fully evaluate its effect on DAPK and apoptosis. In combination with 6-hr exposure to shear stress (12 dynes/cm^2^), we used TNFα (25 ng/ml), H_2_O_2_ (0.5 mM), and serum depletion (0.5% FBS) to induce apoptosis in an 18-hr treatment. We will apply the same 24 hour time frame to all groups to capture apoptotic and DAPK results (Figure 1A).

This study displays similar modulation of DAPK and apoptosis by shear stress with other pro-apoptotic stimulants such as H_2_O_2_ and low serum levels. Onset of shearing single-handedly increased DAPK and apoptosis, but over time shear stress in fact regulates DAPK expression and functions to attenuate apoptosis. We found that pre-treating cells with shear stress significantly decreased DAPK expression and downstream caspase 3/7 activation in the presence of apoptotic activators. Likewise, shearing cells after apoptosis induction decreased DAPK activity and mitigate the apoptotic effect of each apoptosis trigger. Changes in DAPK and caspase 3/7 are directly correlated to changes in apoptosis. Thus, our data suggest that exposure to shear stress mitigates the apoptotic effect of TNFα, H_2_O_2_, and serum depletion through endothelial DAPK expression and caspase activity compared to the static cases. Comparing the effect of different apoptotic triggers, oxidative stress via addition of H_2_O_2_ seemed to induce the greatest increase in DAPK expression, caspase activity, and apoptosis. Regardless of apoptosis trigger, shear stress had a similar attenuating effect on apoptosis either before or after induction. Shear stress applied to cells prior to induction with apoptosis agents resulted in a higher suppression of apoptosis and DAPK and caspase activity, compared to applying shear stress post induction. The difference may be due to an increase in cell quiescence and survival signaling prior to apoptosis induction in pre-sheared cells [33,34], whereas post-sheared cells may already be affected by stimulant exposure prior to shearing.

We also gained more insight into the long term effect of shear stress, independent of any apoptosis factors. Our “6 hr Pre-shear” samples were maintained for 18 hrs in static condition after shearing, before samples were collected for analysis. For “6 hr Post-shear”, samples were maintained under normal static condition, sheared for 6 hrs, then collected for analysis. To evaluate the effect of shearing alone, we compared only the first set of two bars in each of Figure 2, 3, 4 and 5. The “6 hr Post-shear” showed significantly increased DAPK expression and activity, as well as apoptosis, compared to the “6 hr Pre-shear”, probably because cells are collected right after shear stress. For pre-shear vs post-shear cells, there was an increase in DAPK expression and increase in apoptosis; plus a drop in phosphor-DAPK fraction, indicating increased DAPK activity in post-shear. Although, Caspase 3/7 results show a similar increase between pre- and post-shear cells.

Thus, data on the effect of pre- or post-shear alone, without any apoptotic stimulus, suggest a direct link between shear regulated DAPK expression and activity to apoptosis. Because cells are collected right after shearing in “6 hr Post-shear” cells, DAPK showed higher expression and activity, and there’s a corresponding higher level of apoptosis. The exception is caspase 3/7 activity, which is not directly linked to DAPK expression, but is downstream of DAPK expression. In this case, either shearing cells before or after apoptosis induction yielded similar level of caspase activity, though both are significantly increased compared to static cells.

Likewise, this time-dependent shear stress activation of DAPK could also explain its role in different apoptosis pathways. In examining the rest of the data in Figures 2, 3, 4 and 5, the general trend shows shear applied before the addition of apoptotic trigger, rather than after, was more effective in suppressing apoptosis. This could be explained by the opposite time-dependent effect on DAPK, when increasing DAPK expression and activity were observed in “6 hr Post-shear” relative to “6 hr Pre-shear.” It is possible that shear stress on cells after 18 hr apoptosis treatment had a higher, more immediate effect of increasing DAPK expression, which led to higher level of apoptosis. Therefore shear stress in the post-shear cases did not seem as effective in suppressing apoptosis, in part due to the direct increase in DAPK.

The same trend was observed in the order-dependent difference in shear stress suppression of capase 3/7 activity: shear stress applied before treatment with apoptotic stimulus was more effective in suppressing caspase activity. Again this could also be attributed in part to the DAPK increase in cells sheared after apoptotic stimulus, and DAPK is an upstream activator of caspase activity in the apoptotic pathways. Moreover, the effects of a 6 hr exposure to shear stress alone lingered even after 18 hr incubation, and were still statistically significant compared to control cells, shown in the “6 hr Pre-shear” case for Figures 2, 3, 4, and 5. Therefore, cells had a more immediate response to shear stress in activating DAPK and apoptosis, which was also sustained after another 18 hours, compared to static cells.

Endothelial cell reaction to shear stress is seen immediately, i.e. release of nitric oxide, NO [35], in the short-term, such as with tissue plasminogen activator (tPA) gene up-modulation [36]; and also long-term shear stress leads to endothelial cell alignment in the fluid flow direction and to changes in the physical organization, mechanical characteristics, and specific nuclear gene activation [27,37,38]. Endothelial mechanotransduction consist of several signaling constituents such as transcription factors, heat shock proteins, PI3 and mitogen-associated protein (MAP) kinases [39-41]. MAPKs such as JNK, ERK and p38 play a role in endothelial mechanotransduction and directly interact with DAPK in its apoptotic and cytoskeletal association. All three are activated by shear stress at the onset, along with increase in intracellular calcium [42]. ERK is upstream of DAPK activation via phosphorylation at Ser735 [43], phospho-p38 is an apoptotic binding partner of DAPK [44]; and JNK is downstream of DAPK via PKD regulation [45]. As we have discussed before [46], DAPK is involved in multiple apoptotic pathways including cytokine (TNFα), oxidative stress (H_2_O_2_), or serum depletion. Our data support the theory that shear stress may target DAPK as a converging point to exert its effects of suppressing endothelial apoptosis. Regulation of DAPK expression and activity by shear stress closely mirror the effect on apoptosis.

Endothelial cells exhibit cytoskeletal changes: stress fiber formation, focal adhesion rearranging, and re-alignment in the direction of flow after extended periods of shearing (hours) [28]. DAPK is localized to the actin cytoskeleton, stimulates acto-myosin contractility and stabilizes stress fibers in serum-starved fibroblasts through myosin regulatory light chain (MLC) phosphorylation [24,47]. In endothelial cells, DAPK phosphorylates tropomyosin-1 (TM-1) at Ser283 in response to ERK activation under oxidative stress [48]. Thus, DAPK has other non-apoptotic functions in endothelial cells, for example in affecting cell morphology. A novel role for DAPK in the atherosclerosis signaling pathway may provide a therapeutic target for future mechanotransduction studies. We continue to investigate the molecular responses of mechanotransduction and the role of DAPK in endothelial apoptosis.

## Conclusions

In summary, we confirm that exposure to shear stress (12 dynes/cm^2^ for 6 hrs) suppressed endothelial apoptosis triggered by cytokine (TNFα), oxidative stress (H_2_O_2_), and serum depletion, either before or after a long term (18 hr) induction. This correlates with a parallel decrease of DAPK activity and caspase activation when compared to non-sheared cells. Shear stress applied prior to apoptosis induction resulted in a higher suppression of apoptosis, DAPK expression, and caspase activity, as opposed to applying shear stress post induction. This is correlated with a higher expression and activation of DAPK in cells sheared at the end of 24-hr experiment. Also, shear stress alone induced higher apoptosis and DAPK expression, and the effect is sustained even after 18 hrs incubation in static condition, compared to non-sheared cells.

Overall, we show that laminar shear stress deters apoptosis pathways by modulating DAPK expression and activation, as well as decreased caspase activity. The role of DAPK and its mechanotransduction role in shear stress and apoptosis induction are important in fully understanding how cells respond to their local mechanical and chemical environments.

## Methods

### Cell culture

Bovine aortic endothelial cells (BAEC; Lonza) were cultured using Dulbecco’s Modified Eagle Medium (DMEM) with 10% fetal bovine serum (JR Scientific), 1% L-glutamine, and 2% penicillin streptomycin (Sigma), in tissue culture flasks at 37°C with 5% CO_2_. BAEC at passage number 6 to 12 were passed and plated on 38×75×1 mm glass slides at approximately 500,000 cells per slide (for shear stress experiments) and cultured for 24 hours to confluency before experiments. Human TNFα (BD Biosciences) was reconstituted in water to a stock concentration of 10 μg/ml and added to static or shearing media for final working concentration of 10 or 25 ng/ml. Hydrogen peroxide (H_2_O_2_, Sigma) was diluted to a 750 mM stock concentration and added to static or shearing media for a final working concentration of 0.3-0.5 mM. For serum depletion, cell media for static and shearing conditions was the same as previously described except decreased 0.5% FBS to induce apoptosis conditions.

### Flow chamber set-up

Cells were plated on glass slides and placed in parallel plate flow chambers attached to a sterile, laminar flow system in an environmental chamber kept at 37°C with 5% CO_2_. The magnitude of shear stress (τ) on the cell monolayer is calculated based on the Navier–Stokes equation for a Newtonian fluid in a parallel plate geometry. The equation for wall shear stress simplifies to: *τ* = 6*μQ*/(*bh*^2^), where *μ* is the viscosity of the media (0.01 dynes-sec/cm^2^), *Q* is the volumetric flow rate (~0.5 ml/s), *b* is the width of the flow chamber (2.5 cm), and *h* is the separation distance between the chamber and the glass slide (0.027 cm). Using this system, cells were exposed to 12 dynes/cm^2^ laminar wall shear stress. Flow experiments were done using regular growth media. Each of the stimuli could be added to flow using a syringe without interruption. For some experiments, cells were sheared in regular media without phenol red, to reduce background fluorescence.

### Immunoblot analysis

Cells were scraped off slides after experiments and lysed with RIPA buffer with 0.5 mM PMSF, 150 mM protease inhibitor, 1 mM DTT, plus 50 μM sodium fluoride to preserve phosphorylated DAPK. Protein concentrations were measured using the colorimetric Bradford assay. Gel electrophoresis was done using NuPage 4-12% Bis-Tris SDS-PAGE gels (Invitrogen) loaded with equal sample protein amounts in each well, per manufacturer’s instruction. Gels were transferred to 0.45 μm nitrocellulose membrane (GE Technologies). After blocking for 1 hour, anti-DAPK 55 and anti-phospho-DAPK PS308 mouse antibodies (both Sigma) were used to detect protein expression at 1:1000 dilution, followed by goat anti-mouse HRP-conjugate secondary antibody (Bio-Rad) at 1:4000 dilution. Loading control was done using rabbit anti-actin antibody (Sigma) at 1:5000 dilution, followed by goat anti-rabbit HRP-conjugate secondary antibody (Bio-Rad) at 1:4000. All blocking and antibody incubations were done in 5% milk solution made with non-fat dry milk (Carnation) in PBS with 0.1% Tween (PBS-T) at room temperature. Membranes were illuminated using SuperSignal West Pico ECL reagents (Pierce). Imaging was done using BioRad Molecular Imager ChemiDoc XRS+ System. Quantity One Image Analysis Software was used to quantitatively analyze band intensities.

### Flow cytometry

TUNEL stained cells were all quantified using flow cytometry on suspended cells. After experiments, adherent cells on slides were detached gently using non-enzymatic Cell Stripper (CellgroQ14 Mediatech) and fixed with 1% paraformaldehyde and permeabilized with 0.2% Triton X-100 before TUNEL staining using In Situ Cell Death Detection Kit. After TUNEL staining, RNAse A (Roche) and Propidium Iodide (Roche) were used to stain the nucleic acids of the cells. DNAse I (Roche) was used to initiate DNA strand breaks and simulate apoptosis for a positive control sample to set the flow cytometry quadrants. The FACScan flow cytometer (BD Biosciences) was used to categorize cells based on fluorescence characteristics, and CellQuest Pro software was used for data analysis.

### Caspase assay

Cells were plated on glass slides at approximately 500,000 cells per slide 1 day prior to experiments. Afterwards, cells were trypsinated and counted for each sample set, and adjusted to 15,000 cells per 50 μl of culture media. Caspase assay was carried out as per manufacturer’s protocol. Briefly, equal volumes (50 μl) of Caspase Glo 3/7 Reagent (Promega) and cell suspension were combined, mixed gently, and added to white-walled 96-well plates. Blank wells containing media only were used as negative controls against control BAEC and treated cells (Static + TNFα, 6 hr shear + TNFα, and 6 hr shear only). After incubation for 1 hour at room temperature, luminescence of each well was read using a luminometer (PerkinElmer). Each sample was done in triplicates, and averaged values were corrected for media only background readings, and normalized to control samples.

### Statistics

All experimental results were done in triplicates at least. Standard error of each group was calculated to verify the statistical significance of the results. For protein analysis, each sample was normalized to a control standard which serves as a baseline. Quantification of digital band images was done to incorporate repeated blotting results and generate statistical analysis. Each Western band is corrected for background and the loading control on the same blot. Student *t*-test was used to compare one condition between two sample sets. *P* values less than 0.05 was considered sufficient for statistical significance.
